# Brush Cytology with Immunocytochemical Evaluation of VEGF Expression versus Biopsy in Clinically Precancerous Laryngeal Lesions: Can We Diagnose Laryngeal Cancer Only with Brush Cytology?

**DOI:** 10.1155/2015/256182

**Published:** 2015-09-17

**Authors:** Angelos Chatziavramidis, Zinovia Tsinaslanidou, Rozalia Valeri, Iordanis Konstantinidis, Jannis Constantinidis

**Affiliations:** ^1^2nd Academic ORL Department, Aristotle University, Papageorgiou Hospital, Ring Road, Efkarpia, 56403 Thessaloniki, Greece; ^2^Department of Cytopathology, “Theagenion” Oncologic Hospital, Alexandros Symeonidis Street 2, 54007 Thessaloniki, Greece

## Abstract

*Introduction*. Laryngeal squamous cell carcinoma (LSCC) is the most common SCC of the head and neck. The high incidence of this malignancy and the low survival rate necessitate the development of novel diagnostic approaches. Aim of this study is to compare the diagnostic value of laryngeal brush cytology combined with VEGF immunocytochemistry versus histopathology of clinically precancerous lesions of the larynx. *Material and Methods*. Thirty patients with precancerous or suspected malignant laryngeal lesions underwent microlaryngoscopy, during which samples were taken for cytological, immunocytochemical, and histological analysis. Cytology and histology results were classified as follows: benign lesions, atypia/moderate dysplasia, and malignancy, whereas the expression of VEGF was evaluated as strong, moderate, weak, and zero expression, based on the percentage of cells stained. *Results*. The cytology results were in accordance with the histology results in 86.7% of the patients. The exfoliative cytology's sensitivity was estimated at 85% and its specificity at 90%. Its positive prognostic value was 94%, while its negative prognostic value was 75%. The additional immunocytochemical evaluation of VEGF expression increased all the noted parameters. *Discussion*. Exfoliative cytology of laryngeal lesions is a minimal-invasive, easily applicable procedure during microlaryngoscopy and reliable in terms of diagnostic value. Under certain conditions it could be held also in local anesthesia. Concurrent immunocytochemical analysis of VEGF expression further enhances its diagnostic value.

## 1. Introduction 

Laryngeal squamous cell carcinoma (LSCC) accounts for over 95% of all laryngeal malignancies and is the most common SCC of the head and neck with the 5-year overall survival rate estimated at approximately 50% [1–4]. The high incidence of this malignancy and the low survival rate underline the necessity for the introduction of more efficient diagnostic approaches that ideally will enable the doctor to do the following: (a) achieve an early diagnosis before the development of more invasive lesions that require more destructive and debilitating surgery, (b) identify biologically aggressive cancers positive for certain biomarkers that are in a higher risk for metastasis, thus in need for a closer follow-up, (c) determine precancerous lesions which prerequisite a specific follow-up strategy, and (d) identify the disease relapses early in order to adjust in time the treatment applied.

Over the years a number of diagnostic approaches have been developed for laryngeal biopsies, the majority of which are held under general anesthesia, thus requiring hospitalization. Due to the relatively low 5-year survival rate of LSCC and focus on health economic issues in most developed countries a new diagnostic approach less invasive, easily applicable but equally efficient is required. The cytological examination of the larynx by exfoliative technique has been described several decades ago, with the first description made by Ayre J. E. in 1954 [5, 6]. Although the exfoliative cytology is regarded as a revelation in the screening of cervical cancer, its use in the larynx has been underestimated for many years. A number of studies have evaluated the application of exfoliative cytology in comparison with the widely applied histological examination in laryngeal lesions presenting results that vary from extremely high to less than 50% [5, 7–13].

The expression of angiogenic factors in laryngeal cancer is under the microscope providing some insight into the prognosis of the disease and moreover serving as a target for the development of antiangiogenic therapeutic approaches [2, 14]. Vascular Endothelial Growth Factor (VEGF) is the most widely studied angiogenic factor. This pivotal molecule is produced by tumor and stromal cells and acts via its receptors (VEGFRs), present in endothelial and tumor cells, promoting angiogenesis, resulting in tumor growth [15]. Although the expression of VEGF in laryngeal cancer histopathology samples has been thoroughly studied, the molecular analysis of its expression in cytologic samples has not received adequate attention. The ability to perform immunocytochemical analysis on the cytology samples in order to examine the expression of certain agents such as Proliferating Cell Nuclear Agent (PCNA), Ki-67, and VEGF is expected to increase further the accuracy of the cytology examination in terms of diagnosis, embedding it in the armamentarium of the otorhinolaryngologist.

In our study we correlate the results of exfoliative cytology application on precancerous or suspected malignant laryngeal lesions during microlaryngoscopy with concurrent immunocytochemical analysis for VEGF expression with the results of the histological analysis performed on the same lesions.

## 2. Materials and Methods

It was a prospective, nonrandomized clinical study. The prerequisite for the patients in order to be included in our study was the presence of a precancerous or suspected malignant laryngeal lesion. Exclusion criteria were an oncologic history with recent chemotherapy or head and neck radiation, pregnancy, and systemic diseases that may increase the risk of complications during surgery (heart disease, pulmonary disease, chronic renal/liver insufficiency, etc.). Moreover, patients that could not comply with the follow-up appointments that our protocol required were excluded. The first 30 patients administered in our tertiary academic ENT department during an 18-month period, who met the inclusion and exclusion criteria, were enrolled in our study. The study was approved by the hospital ethics committee and was carried out in accordance with the Declaration of Helsinki. All patients were thoroughly informed about the clinical protocol and gave written consent. The baseline characteristics of the patients that were enrolled in the study are outlined in Table 1.

In all patients cytological, immunocytochemical, and histological examinations were performed. After the insertion of the laryngoscope and before the surgeon proceeded to any intervention on the lesion, a sample for cytology examination was taken from the “suspicious” area via a cytology brush. The brush was placed upon the lesion and then it was rotated through 360° and concomitantly moved along the sagittal axis of the lesion, until pinpoint bleeding appeared in order to obtain a transepithelial cytology sample. The trapped exfoliated cells were smeared on two coded glass slides and were immediately fixed with the use of fixative spray (conventional exfoliative cytology). Care was taken not to squeeze the material during this process. Then, the head of the brush was separated from the body and was immersed into a cytofixative solution for liquid based cytology to be performed (ThinPrep Cytyc Co., USA). This is an automated method of specimen preparation and distribution of cells in a thin, evenly dispersed layer. Afterwards, a conventional biopsy of the lesion was performed and the extracted tissue was immersed into a formalin solution. The expression of VEGF was examined in the cytology samples immunocytochemically with the use of VEGF monoclonal antibodies (clone VG1, Diagnostic Biosystems, Ylem). All specimens were examined by the same cytologist and histopathologist both were ignorant of each other's results.

The cytology examination results were classified as follows: (a) benign lesion, (b) atypical/moderate dysplastic lesion (suspicious lesion), and (c) malignant lesion. The same classification was additionally followed for the results of the histological examination. Furthermore cytoplasmic and cell membrane staining for VEGF was evaluated. VEGF expression of the lesion was evaluated from 0 to 3 in terms of the percentage of VEGF expressing cells per low-power field. The staining positivity was scored as strong (3+ when >50% of cells were stained), moderate (2+ when 15–50% of cells were stained), weak (1+ when <15% of cells were stained), and 0 (when none of the cells were stained). We examined the correlation between the exfoliative cytology with concurrent immunocytochemical analysis with the histologic results, which were considered as diagnostically correct, thus defining the following therapeutic approach. For all patients an 18-month follow-up was scheduled, in order to evaluate the clinical course of the disease and to confirm the primary diagnosis.

## 3. Results

During a period of 18 months, 30 patients underwent microlaryngoscopy under general anesthesia for precancerous or suspected malignant laryngeal lesions. During the procedure samples were taken for both cytologic and histopathologic examination. Macroscopically, the lesions were characterized as leukoplakias in 20 patients (66.7%) and erythroplakias in 5 patients (16.6%), whereas in 5 patients (16.6%) the lesions were highly suspected for malignancy. All procedures were completed uneventfully.

**Figure 1:**
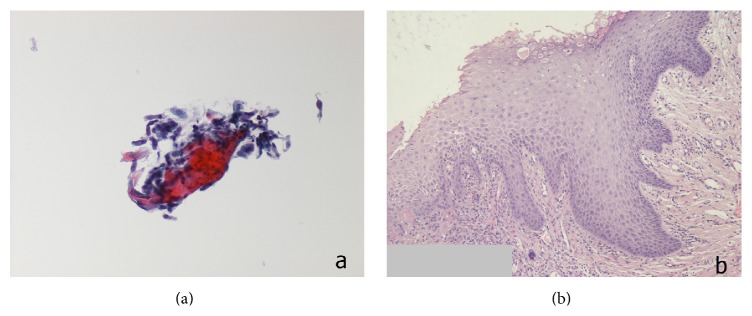
(a) Cytology-squamous cells (ThinPrep; Pap stain ×400). (b) Histology-hyperplastic squamous epithelium; Haematoxylin & Eosin ×200.

**Figure 2:**
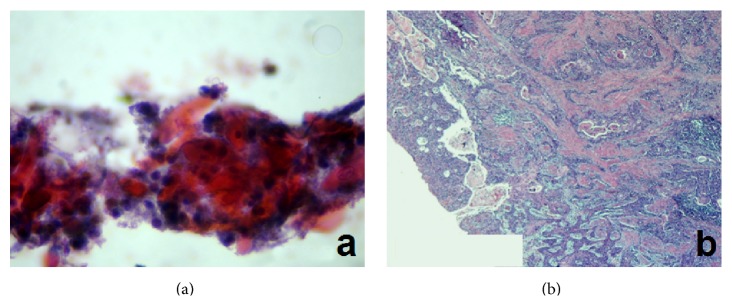
(a) Cytology-mild dysplasia (ThinPrep; Pap stain ×400). (b) Laryngeal mucosa with infiltrated chromium by squamous cell carcinoma of moderate differentiation. Cellular and nuclear polymorphia and increased number of mitoses; Haematoxylin & Eosin ×40.

In 26 patients (86.7%) the results of the exfoliative cytology were in accordance with those of the histologic examination (Figure 1). More specifically, 17 patients were diagnosed with laryngeal malignancy, 4 with atypia/moderate dysplasia (suspicious lesion), and 5 with benign lesions. In 4 patients the cytologic examination results opposed the histologic analysis (Figure 2). The exfoliative cytology diagnosed atypia/moderate dysplasia (suspicious lesion) in 3 patients, whereas the histological examination identified the laryngeal lesion as malignant (in situ, microinvasive, or invasive SCC). Additionally, in one patient, the lesion was cytologically classified as malignancy, while, on the contrary, the histologic examination diagnosed atypia/moderate dysplasia (suspicious lesion). In this patient, due to clinical suspicion of malignancy, a revision biopsy was performed, on which the new histological examination confirmed the absence of malignancy. The correlation between the results of the cytologic and histologic examination is summarized in Table 2.

**Figure 3:**
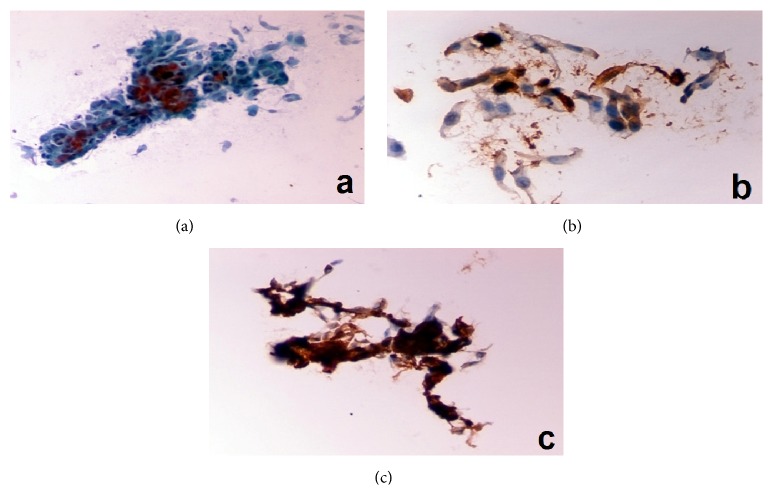
(a) Cytology-SCC (Pap stain, conventional ×400). (b) Immunocytochemistry-SCC with VEGF expression 2+ (ThinPrep ×400). (c) SCC with VEGF expression 3+ (ThinPrep ×600).

In all 5 lesions that were classified by cytologic analysis as benign no VEGF expression was noted. From the 7 patients diagnosed as atypia/moderate dysplasia (suspicious lesion) by exfoliative cytology, 5 showed high or moderate VEGF expression (classified as 2 or 3) and 1 showed weak expression (classified as 1), whereas there was one patient not expressing the antibody at all (classified as 0). On the other hand, in the 18 patients where malignancy was diagnosed, VEGF expression was noted as high or moderate in 10 cases (classified as 2 or 3) (Figure 3), weak in 4 cases (classified as 1), and zero expression in 4 cases (classified as 0). The VEGF expression in correlation with the results of exfoliative cytology is outlined in Table 3.

In the cases where disagreement was observed between cytology and histopathology examinations' results, attention was drawn in the expression of VEGF. The angiogenic factor was examined as an additional marker that might enhance the accuracy of exfoliative cytology. The 3 patients were diagnosed cytologically as atypia/moderate dysplasia (suspicious lesion), whereas the histologic examination revealed malignancy, showing high or moderate expression of VEGF (specifically 2 were classified as 3+ and one was classified as 2+). On the other hand, in the 4 cases diagnosed both cytologically and histologically as atypia/moderate dysplasia (suspicious lesion), VEGF expression was classified as 3+ in one case, 2+ in another case, and 1+ in the last case. In the patient that was diagnosed histologically as atypia/moderate dysplasia, while cytology stated malignancy, the VEGF expression was noted moderate, classified as 2+.

The diagnosis in all cases was based on the results of the histology examination. It must be noted that the concordance between cytological and histopathological results was 86.7% (26/30). Based on the outcome, the sensitivity, specificity, and the accuracy of cytological assessment were evaluated. Sensitivity was estimated at 85%, specificity at 90%, and accuracy at 86.7%.

The positive prognostic value of laryngeal exfoliative cytology was estimated at 94%, while the negative prognostic value was estimated at 75%.

Over the 18-month follow-up period, none of the patients diagnosed with benign lesions or atypia/moderate dysplasia (suspicious lesions) developed malignancy, requiring further treatment.

## 4. Discussion

The incidence of laryngeal cancer has increased rapidly over the last decades, with the squamous cell carcinoma being the most common laryngeal malignancy. LSCC accounts for more than 95% of all laryngeal malignancies. It represents 10% of malignancies in men and 4% in women, with its highest incidence observed between the 5th and 6th decades of life [1, 2]. The outcome in patients diagnosed with LSCC is determined by the stage of the disease at the time of diagnosis and the site of the tumor. Therefore, early diagnosis is the cornerstone of the treatment strategies [3].

In 2005, WHO first introduced the term “squamous intraepithelial neoplasia, (SIN)” for the precancerous lesions, which describes the extent of cytological and architectural abnormalities in squamous epithelium with no penetration of the basement membrane. It is graded as SIN-1, SIN-2, and SIN-3 on the basis of the degree of nuclear abnormalities as well as the level of epithelium showing loss of stratification [16].

Cytologic examination of the larynx has been described several decades ago. Despite the fact that exfoliative cytology with Pap-smear examination exhibiting a sensitivity of roughly 60% [17] is regarded as a revelation in the screening of uterine cervical cancer, its application on laryngeal lesions has been limited over the years. The main disadvantage of laryngeal cytology is its inability to evaluate the basement membrane, thus making the differential diagnosis between high-grade dysplasia, in situ carcinoma, and invasive cancer difficult. Therefore, it is impossible to achieve the same classification that the histological examination of the laryngeal lesions provides. The cytologic examination can categorize the lesions as benign lesions, laryngeal intraepithelial neoplasia (LIN), and malignancy. In order to amplify its diagnostic accuracy in laryngeal lesions, a transepithelial cytology sample must be obtained. This is achieved by rotating the biopsy brush along the laryngeal lesion, until pinpoint bleeding appears, an approach that has been used in our study as well.

In the early stage laryngeal lesions, false negative histologic results are not uncommon, requiring revision biopsies of the vocal cords, which can account for functional impairment. The exfoliative cytology can contribute to diagnosis in such cases as it is non-/minimal-invasive and could be applied under local anesthesia on an outpatient basis. It has the advantage of allowing sampling from an area of the laryngeal epithelium larger than the macroscopically present lesion, in accordance with the “field cancerization theory,” without significant damage of the surrounding tissues [18–20]. Taking into consideration the fact that incidence of LSCC in lesions macroscopically characterized as leukoplakias or erythroplakias is relatively low (5% and 25%, resp.), the indication of a biopsy during a microlaryngoscopy procedure must be placed after careful evaluation of the patient, as it is held under general anesthesia and has a number of complications [21]. It is also known that an epithelial hyperplastic lesion of the larynx may gradually degenerate to dysplasia or even carcinoma in situ, not necessarily passing from all the intermittent stages of dysplasia [16]. Consequently, exfoliative cytology of precancerous laryngeal lesions could be used as a first-line diagnostic (screening) tool, in order to evaluate those lesions in which a further biopsy during microlaryngoscopy is required. In terms of health economics the application of exfoliative cytology could minimize the number of microlaryngoscopies required in order to evaluate precancerous or suspected malignant laryngeal lesions improving also the microlaryngoscopy waiting list time for those cases showing an urgent character.

In our study, we evaluated the results of cytologic examination of precancerous or suspected malignant lesions under general anesthesia prior to biopsy with concurrent immunocytochemical analysis for VEGF expression in correlation with the results of the histological analysis performed on the same lesions. The PPV and the NPV of exfoliative cytology were 94% and 75%, respectively, with the sensitivity evaluated at 85%, specificity at 90%, and accuracy at 86.7%. The review of the literature reveals a number of studies that favor the application of laryngeal cytology. Malamou-Mitsi et al. estimated its sensitivity at 93.3%, its specificity at 100%, and its accuracy at 96.7% [11]. The same parameters were evaluated by Ustundag et al. at 82.5%, 94.5%, and 87.4%, respectively, whereas Lundgren et al. estimated sensitivity at 83% and specificity at 84%, respectively, and Gugatschka et al. raised sensitivity up to 97% when the cytology was applied in combination with stroboscopy [3, 10, 13]. On the other hand, previous studies, for example, the one of Thomsen et al., with 44% false negative results, concluded that the cytology examination is not appropriate as a screening method for laryngeal lesions [22].

These conflicting results may be due to different approaches in obtaining the cytology sample (use of cotton stick instead of brush or spatula, transepithelial or superficial sample, etc.) and due to different processing methods of the cytological material itself. As Thompsen et al. mentioned in their article “it is not difficult to make a laryngeal smear during indirect laryngoscopy if the patient has a good local anaesthesia; but one must be gentle. This is the reason for using only a cotton stick for obtaining a cytological smear.”

In our study, the cytological material from the laryngeal lesions was processed using both conventional and liquid based cytology (ThinPrep technique). Liquid based cytology offers automated or semiautomated processing and distribution of cells in a thin, evenly dispersed layer, to enhance specificity and sensitivity. The method is simple, not of great cost, and permits faster and better evaluation of the smear [23]. The great advantage, over conventional method, is that it gives the possibility of creating archive material and applying new techniques, as immunocytochemistry, on the same sample [24]. The application of immunocytochemistry on liquid based cytology smears, especially ThinPrep, as compared to the conventional method, has facilitated its use and provided additional benefits, increasing further its sensitivity, specificity, and accuracy.

Additionally, immunocytochemical analysis of the cytology samples in order to examine the expression of certain angiogenic agents is expected to enhance further the sensitivity, specificity, and accuracy of laryngeal cytology. The expression of angiogenic factors in LSCC has been studied over the last decades in histological samples serving as a prognostic factor of the disease progression and as a target for development of antiangiogenic therapeutic approaches [2, 14]. VEGF is the most studied angiogenic factor. VEGF gene is located on the sixth chromosome, encoding a heparin-binding glycoprotein with at least four molecular forms [25]. VEGF expression is upregulated in LSCC, as in most human malignancies, showing increased expression with progression from mild to moderate, to severe dysplasia, and finally to carcinoma [2]. VEGF has mitogenic activity specific for endothelial cell and is implicated in the angiogenesis process associated with tumors. Pentheroudakis et al. (2012) have shown that VEGF overexpression has been associated with a 2-fold higher risk of death at 2 years in a meta-analysis of 12 studies comprising 1002 patients with HNSCC [15]. Moreover, high VEGF expression correlates with a higher rate of disease recurrence, especially distant recurrences, and shorter progression-free intervals [4]. On the other hand, none to low expression predicts a high probability of obtaining complete remissions with induction chemotherapy, whereas no such correlation was noticed in terms of radiotherapy outcomes [26]. The review of the literature outlines several studies reaching results that at first sight appear to be controversial as far as VEGF expression and LSCC correlation. These differences can be attributed to different methods of detection of VEGF expression, such as immunohistochemistry, ELISA, and RT-PCR [1, 4]. Our study results reveal a correlation between VEGF expression levels and the extent of cytological and architectural abnormalities in LSCC, in terms of benign lesions, atypia/moderate dysplasia, and malignancy. In 5 out of 7 patients with suspicious cytology the expression of VEGF was estimated as moderate/high while one had weak and the other one had zero VEGF. This correlation between cytologically suspicious lesions and VEGF immunocytochemistry enhances the diagnostic accuracy of laryngeal cytology especially regarding these borderline lesions. The group of 18 patients with cytologically malignant lesions had in 14 cases positive results for VEGF (10 with moderate/high and 4 with weak expression) and 4 with negative results. From the above data we assume that the expression of VEGF is an adjunctive argument for the minimization of false positive results. Although no statistically significant results have been reached due to the small number of patients, the estimation of VEGF expression in laryngeal precancerous or suspected malignant lesions can be a valuable adjunct to laryngeal cytology.

In order to increase the diagnostic accuracy of exfoliative laryngeal cytology further improvements in the sampling technique must be made, the close cooperation between the ENT doctor and the cytopathologists must be guaranteed, and further specialization of cytopathologist in laryngeal cytology should be encouraged. Additionally, the development of new antibodies in immunohistochemistry is expected to enhance the diagnostic value of cytopathology.

## 5. Conclusions

Exfoliative cytology of laryngeal lesions is a minimal-invasive, time-efficient, and reliable method in terms of diagnostic accuracy that could be performed in an outpatient basis. The use of immunocytochemical analysis in cytology samples in order to estimate VEGF expression enhances additionally the diagnostic value of laryngeal cytology. One must underline the fact that exfoliative cytology and histology examination are not competitive diagnostic procedures. On the contrary, laryngeal cytology is a valuable adjunct to histology. The positive correlation between the two diagnostic procedures justifies the use of cytology examination as a first-line diagnostic tool in both primary diagnosis and follow-up examinations.

## Figures and Tables

**Table 1:** Baseline characteristics of the patients enrolled in the study.

Patient data	*N* (%)
Sex	
Male	28 (73.3%)
Female	2 (6.7%)
Age	55.7 y.o. (38–73 y.o.)
Macroscopic evaluation of the lesion	
Leukoplakia	20 (66.7%)
Erythroplakia	5 (16.6%)
Suspected malignancy	5 (16.6%)

**Table 2:** Cross-table of the correlation between the results of the cytologic and histologic examination of 30 patients with precancerous or suspected malignant laryngeal lesions.

Histologic examination	Exfoliative cytology examination		
	*Malignancy*	Benign lesions	Atypia/moderate dysplasia (suspicious)
*Malignancy*	17	—	3
Benign lesions	—	5	—
Atypia/moderate dysplasia	1^*∗*^	—	4

^*∗*^The patient underwent a revision biopsy.

**Table 3:** The correlation between the results of exfoliative cytology and the immunocytochemical analysis of VEGF expression in 30 patients with precancerous or suspected malignant laryngeal lesions.

	VEGF expression	0	1	2	3
Cytologic examination	Benign lesions	5	—	—	—
	Atypia/moderate dysplasia (suspicious)	1	1	2	3
	*Malignancy*	4	4	4	6
